# Janzen’s Hypothesis Meets the Bogert Effect: Connecting Climate Variation, Thermoregulatory Behavior, and Rates of Physiological Evolution

**DOI:** 10.1093/iob/oby002

**Published:** 2019-01-02

**Authors:** M M Muñoz, B L Bodensteiner

**Affiliations:** Department of Biological Sciences, Virginia Tech, Blacksburg, VA 24060

## Abstract

Understanding the motors and brakes that guide physiological evolution is a topic of keen interest, and is of increasing importance in light of global climate change. For more than half a century, Janzen’s hypothesis has been used to understand how climatic variability influences physiological divergence across elevation and latitude. At the same time, there has been increasing recognition that behavior and physiological evolution are mechanistically linked, with regulatory behaviors often serving to dampen environmental selection and stymie evolution (a phenomenon termed the Bogert effect). Here, we illustrate how some aspects of Janzen’s hypothesis and the Bogert effect can be connected to conceptually link climate, behavior, and rates of physiological evolution in a common framework. First, we demonstrate how thermal heterogeneity varies between nighttime and daytime environments across elevation in a tropical mountain. Using data from Hispaniolan *Anolis* lizards, we show how clinal variation in cold tolerance is consistent with thermally homogenous nighttime environments. Elevational patterns of heat tolerance and the preferred temperature, in contrast, are best explained by incorporating the buffering effects of thermoregulatory behavior in thermally heterogeneous daytime environments. In turn, climatic variation and behavior interact to determine rates of physiological evolution, with heat tolerance and the preferred temperature evolving much more slowly than cold tolerance. Conceptually bridging some aspects of Janzen’s hypothesis and the Bogert effect provides an integrative, cohesive framework illustrating how environment and behavior interact to shape patterns of physiological evolution.

## Introduction

Discovering the guiding principles that predictably link climate to physiological adaptation and the evolution of biodiversity is an enduring goal in biology (Andrewartha and Birch 1954; Spicer and Gaston 1999; Erwin 2009). As current global climate change marches on, this goal has become especially important for determining how rapidly changing environments will impact organisms (e.g., Williams et al. 2008; Bellard et al. 2012; Huey et al. 2012; Moritz and Agudo 2013; Root et al. 2015; Muñoz and Moritz 2016). One of the central, unifying syntheses in this realm is Janzen’s (1967) treatise titled, “Why Mountain Passes are Higher in the Tropics,” which encompasses a set of ideas commonly referred to as the “climate variability hypothesis” or, simply, “Janzen’s hypothesis” (discussed in Ghalambor et al. 2006; Sheldon et al. 2018). Janzen’s key advance was to create a mechanistic link climatic variation across elevation and latitude, physiological adaptation, and population demography in a single, synthetic framework. As he predicted, climatic variation is indeed an extremely strong and, often, sufficient predictor of large-scale geographic patterns of physiological variation across temperate and tropical landscapes (Pearson and Dawson 2003; Currie et al. 2004; Sunday et al. 2011).

In recent years, it has also become increasingly clear that organismal behavior (and its interactions with climatic variation) is an equally important factor structuring evolutionary patterns in physiology and shaping potential responses to contemporary climate change (Huey 1991; Angilletta et al. 2002; Huey et al. 2003, 2012; Deutsch et al. 2008; Muñoz et al. 2014, 2016). Behavioral thermoregulation occurs when an organism behaviorally maintains a relatively stable core temperature. In an evolutionary context, organisms can use behavioral thermoregulation to erode (or even erase) environmental selection on physiology and homogenize the effects of climatic variation. As a consequence, thermoregulation—or, more broadly, any regulatory behavior—can preclude physiological evolution even in the face of changing climatic conditions, a phenomenon commonly referred to as the “Bogert effect” (Huey et al. 2003).

Both Janzen’s hypothesis and the Bogert effect have received robust theoretical and empirical support and are important and useful concepts (Huey et al. 2003; Ghalambor et al. 2006; Marais and Chown 2008; Muñoz and Losos 2018; Sheldon et al. 2018). These ideas, however, have largely been studied independently. Our goal here is to discuss how jointly considering thermal heterogeneity, behavioral thermoregulation, and physiology can create a broadly predictive framework for rates of trait evolution, with implications for assessing vulnerability to climate change. Specifically, we will show how Janzen’s (1967) conceptual framework can be combined with behavioral data to help understand differences in physiological trait evolution. To illustrate these points, we will give an example using our previous and current work on a group of *Anolis* lizards from the Caribbean island of Hispaniola. We use this example as launching point for a broader discussion connecting climate and behavior in studies of evolutionary physiology.

## Janzen’s hypothesis: thermal resource distributions impact geographic patterns of physiology

**Fig. 1:**
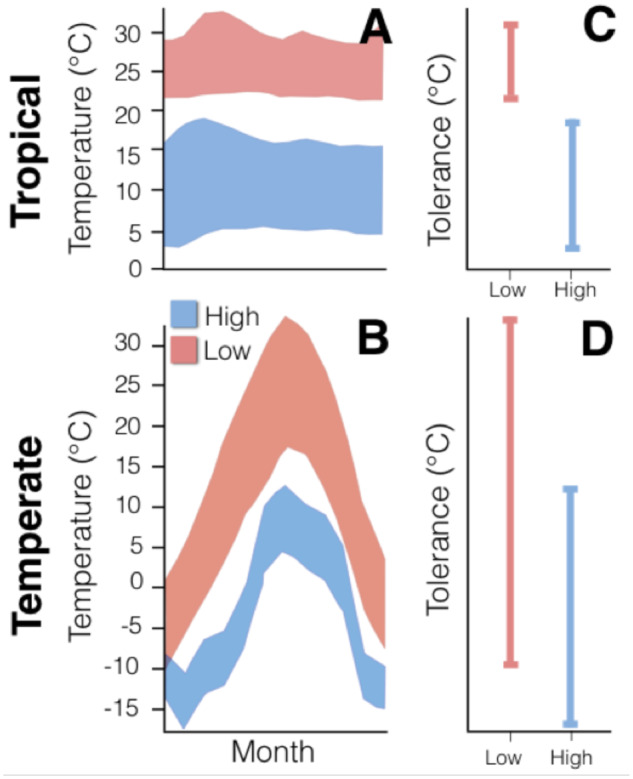
(A and B) Environmental temperature (*y*-axis) is plotted against month (*x*-axis) from January (left) to December (right). Temperatures measured from low elevation are shown in red, while temperatures from high elevation are shown in blue. (C and D) The predicted thermal tolerance range (i.e., the temperature range between the critical thermal minimum and maximum) is given for low elevation taxa in red and for high elevation taxa in blue. (**A**) Low seasonality in tropical habitats results in thermal stability across seasons. Consequently, low elevation (red) and high elevation (blue) sites exhibit little environmental overlap throughout the year. (**B**) High seasonality at temperate latitudes results in greater within-site variation and, consequently, greater thermal overlap throughout the year between low- and high elevation sites. (**C**) Low seasonality in tropical mountains should result in physiological specialization and little physiological overlap across elevation. (**D**) High seasonality in temperate mountains should result in populations composed of generalists, with high physiological overlap across elevation. Panels A and B re-drawn from Janzen (1967).

Janzen’s (1967) hypothesis is premised on a few observations connecting temperature variation and physiological tolerance (discussed in Ghalambor et al. 2006; Sheldon et al. 2018). First, seasonal variation in temperature is much lower in tropical mountains than in temperate mountains (Fig. 1A, B). At any given site in a tropical mountain, ambient temperatures remain relatively stable across seasons, whereas on a temperate mountain, thermal conditions vary considerably more within and across seasons. Because temperature decreases with altitude (MacArthur 1972; Körner 1999; Dillon et al. 2005), tropical mountains can be generally structured into distinct thermal zones, with relatively little thermal overlap between low and high elevation sites across seasons (Fig. 1A). In temperate mountains, in contrast, greater variation among seasons results in more thermal overlap across elevation (Fig. 1B). As a result, both within-site and among site thermal variation should be higher in temperate mountains than in tropical mountains.

Janzen further predicted that species at higher latitudes should exhibit broad thermal tolerances (i.e., the range between the critical thermal minimum [CT_min_] and the critical thermal maximum [CT_max_]; Fig. 1D), which should reflect the broad range of temperatures temperate organisms experience during their lifetimes. In contrast, species from tropical mountains experience more aseasonal thermal environments and should thus be thermally specialized to a narrow range of conditions (Fig. 1C). As a result, physiological tolerances should be broader within populations and overlapping among populations in temperate mountains. In contrast, tolerances should be narrower and less overlapping across elevation in tropical mountains. For an equivalent shift in altitude, tropical organisms will be exposed to more conditions to which they are not well-adapted than temperate organisms. Thus, mountain passes in the tropics are “higher” because of the greater physiological costs associated with the change in environmental conditions, and elevation being a stronger isolating factor in tropical mountains than in temperate mountains (Janzen 1967).

Janzen’s (1967) insights on temperature variation can be extended beyond comparisons across latitudes and seasons. To illustrate this, we will give an example using daytime and nighttime temperatures collected across altitude on the Caribbean island of Hispaniola (Supplementary Fig. S1). Whether at sea level or at high elevation, solar radiation increases daytime temperatures, resulting in a high thermal range that should be largely shared across elevational bands (Sarmiento 1986; Ghalambor et al. 2006). When considered across elevation, the pattern of diurnal temperature variation should, therefore, recapitulate the pattern (though obviously not the range) that Janzen predicted for temperate mountains across seasons (akin to Fig. 1B). At night, in contrast, temperatures should be considerably more constant and progressively cooler with elevation and, therefore, recapitulate the pattern Janzen predicted across seasons in tropical mountains (akin to Fig. 1A).

In June 2013, we visited four sites in the western Dominican Republic that, together, spanned more than 2 km in elevation (45 m, 727 m, 1395 m, and 2318 m; Supplementary Table S1). At each of these four sites, we set out 23 lizard models that each contained an embedded iButton temperature sensor (Supplementary Figs. S1, S2). These lizard models were made from electroformed copper, painted to match the reflectance of *Anolis cybotes*, and calibrated against live lizards (details in Muñoz et al. 2014). Thus, the thermal inertia of the copper lizards was similar to that of live lizards. By being deployed in large numbers, the models provided a realistic null distribution of the steady-state temperatures, or operative temperatures, that lizards would exhibit in the absence of physiological or behavioral regulation (Huey 1991; Bakken 1992; Hertz 1992a). Based on the ecology of the study species (trunk-ground anoles; Schwartz 1989; Losos 2009), we deployed the models onto appropriate lizard perches (boulders, tree trunks, and branches) with perch selection made using a random number generator. The iButton sensors recorded temperature every 10 min during the course of one 24-h period at each site.

**Table 1:** Data from Muñoz et al. (2014), Muñoz and Losos (2018), and supplemented with newly collected data, showing elevation for each population of lizard sampled.

		CT_min_				T_pref_				CT_max_				
	alt. (m)	*n*	mean	variance	range	*n*	mean	variance	range	*n*	mean	variance	range
*Anolis cybotes* (SB)^a^	45	16	11.3	1.9	7	19	28.6	1.7	4.8	20	39.5	0.4	2.5
*Anolis cybotes* (CC)	56	16	11.4	3.6	2.5	13	28.8	14.7	11.2	16	39.2	0.8	3
*Anolis longitibialis*	105	18	12.8	3.1	3.5	12	28.5	6.8	7.4	18	38.5	1.0	4.5
*Anolis whitemani*	411	15	12.2	1.9	4.5					15	38.8	1.4	3.5
*Anolis strahmi*	454	6	11.3	1.6	3					6	39.2	0.2	1
*Anolis marcanoi*	458	9	12.6	2.3	2.5	6	29.4	2.1	3.2	9	38.2	0.6	2.5
*Anolis cybotes* (CC)	690	18	10.7	3.0	5.5	13	28.8	10.1	10.1	18	40.3	3.1	6.5
*Anolis cybotes* (SB)^a^	727	15	10.8	2.2	4.5					15	38.7	0.7	3.5
*Anolis marcanoi*	879	16	11.3	1.7	5.5					16	38.7	0.7	3
*Anolis cybotes* (CC)	1390	11	10.0	2.2	5	15	30.7	13.9	13.9	11	39.5	4.6	6
*Anolis cybotes* (SB)^a^	1395	9	8.7	8.1	4	11	27.5	2.8	5.7	9	38.9	1.6	5
*Anolis shrevei*	1950	9	9.6	3.2	6					9	39.9	1.9	5
*Anolis armouri*	2020	9	8.2	1.4	6								
*Anolis armouri*^a^	2318	12	7.2	1.3	3.5	21	30.5	2.0	4.9	9	39.3	4.2	2.5
*Anolis shrevei*	2450	11	6.2	2.9	6	19	29.3	14.5	12.6	11	40.4	0.6	7

*Notes*: Population means for physiological traits are given, along with sample size (*n*), population variance, and range (i.e., the range of values for each trait within a population). Populations of *Anolis cybotes* with SB in parentheses denote populations from the Sierra de Baoruco mountain chain whereas those with a CC in parentheses come from the Cordillera Central mountain chain.

**Fig. 2:**
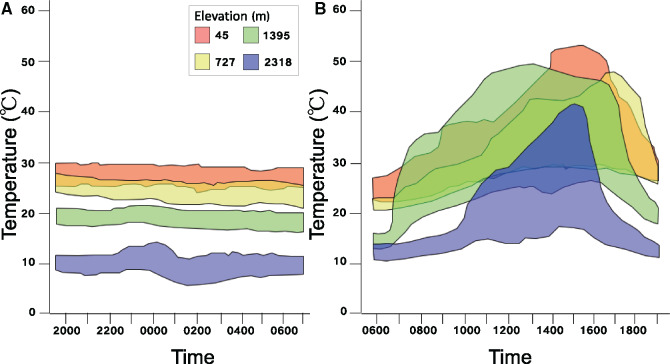
(**A**) Range of nighttime operative temperatures measured at four sites (45, 727, 1395, and 2318 m) in the Sierra de Baroruco mountain range of the Dominican Republic. (**B**) Range of daytime operative temperatures measured at the same sites as (A). Colors correspond to the specific sites, per the legend provided.

a Localities where operative temperature data were taken (data presented in Fig. 2). Coordinates for each locality are supplied in Supplementary Table S1.

Regardless of elevation, operative temperatures were 3–4 times more variable during the day (Fig. 2B) than during the night (Fig. 2A). Importantly, much of that thermal variation was shared across sites, particularly at low- and mid-elevation sites. At high elevation, operative temperatures were considerably cooler, especially during the early morning and late afternoon, than at the other sites. Nonetheless, during the middle of the day, operative temperatures at high elevation were quite warm (often in excess of 30°C) and overlapped substantially with the other three sites. Thus, thermal variation within sites and overlap among elevation follows a similar pattern (though not range) to that observed across seasons on temperate mountains (Fig. 1B). In contrast, nighttime operative temperatures were considerably more stable across all sites, and varied less than 10°C within sites (Fig. 2A). Importantly, little to no thermal variation was shared among sites at night (see lack of color overlap in Fig. 2A). For example, none of the nighttime thermal conditions measured at high elevation (2318 m) were observed at any of the other three sites. Thus, the pattern of low thermal variation within sites and reduced overlap among elevation is similar to the pattern observed across seasons on tropical mountains (Fig. 1A).

Although these operative temperatures were measured over a single day at each site, the general patterns (greater thermal heterogeneity during the day) are well supported by previous work. Operative temperature variation is typically much greater during daytime hours than at night (e.g., Chappell 1981; Dorcas and Peterson 1998; Blouin-Demers and Weatherhead 2001; Anderson et al. 2005), although the disparity in this pattern can shift across seasons (Zimmerman et al. 1994; Kearney and Predavec 2000; Corkery et al. 2018).

## Daily temperature variation and physiological variation

Given that variation in daytime and nighttime operative temperature parallels patterns across seasons and latitudes, Janzen’s ideas might also be extended to supply predictions about patterns of physiological adaptation. To illustrate this point, we use data from published and unpublished work on the thermal physiology of the cybotoid clade of trunk-ground anoles from Hispaniola (Muñoz et al. 2014; Muñoz and Losos 2018). We focus on three physiological traits: cold tolerance (CT_min_), the preferred temperature (T_pref_), and heat tolerance (CT_max_). Cold and heat tolerance refer to the thermal limits for performance, and are typically measured as the lower and upper temperatures, respectively, at which lizards lose the ability to right themselves when flipped on their backs (Spellerberg 1972). The preferred temperature refers to the mean temperature of a lizard that has been put in a thermal gradient and allowed to choose where to sit (Huey 1982; Hertz et al. 1993), and typically correlates strongly with the optimal performance temperature, particularly in diurnal lizards like anoles (Huey et al. 2012).

Cold tolerance is often correlated with the minimum environmental temperatures (or related proxies) that organisms experience (Addo-Bediako et al. 2000; Gibert and Huey 2001; Kimura 2004; Cruz et al. 2005; Calosi et al. 2010; Clusella-Trullas et al. 2011; Kellermann et al. 2012a; Overgaard et al. 2014). We could, therefore, predict that within-population variation for cold tolerance is fairly narrow, with relatively little overlap across elevation (i.e., akin to Fig. 1C). In contrast, upper physiological limits, like T_pref_ and CT_max_, should be expected to experience stronger environmental selection during the day than at night, when temperatures are warmest and more variable. Consequently, we could predict that within-population variation for T_pref_ and CT_max_ should be broader and higher among-population overlap, reflecting the wider range and overlap of daytime environmental temperatures (i.e., akin to Fig. 1D). It is important to note that, in his original formulation, Janzen (1967) was referring to the effects of seasonal temperature variation and on thermal performance breadth (i.e., the range of temperatures over which organisms can move). Here, we are not examining performance range (and, therefore, this is not a test of Janzen’s hypothesis), but rather we are inferring that within-population trait variance should reflect environmental variation. We also note (and discuss later) that minimum and maximum environmental temperatures are certainly not the only factors impacting physiological evolution and that these hypotheses reflect a simplified selective scenario.

Our focal clade, the cybotoid trunk-ground anoles, spans an exceptionally wide range of elevations on Hispaniola (sea level to >2500 m), where lizards occupy habitats ranging from scrubby semi-deserts to montane pine forests (Hertz and Huey 1981; Schwartz 1989; Glor et al. 2003). We present data from 15 populations of lizards (representing seven species) that, together, spanned over 2 km in elevation and included representative populations from both major mountain chains of the island (Cordillera Central and Sierra de Baoruco; Supplementary Table S1). CT_min_ and CT_max_ refer to the thermal limits of performance, which we measured as the lower and upper temperatures, respectively, at which a lizard failed to right itself when flipped onto its back (Spellerberg 1972; Lutterschmidt and Hutchison 1997). Briefly, we exposed lizards to a cold source (ice) or a heat source (heat lamp) and lowered or raised body temperature by 1°C /min. Following established protocols (detailed in Muñoz et al. 2014), we flipped lizards onto their backs and encouraged them to flip over by manual stimulation. We measured the preferred body temperature by placing lizards into a thermal arena (temperature range: 18–40°C), where they were free to move around. As described in Muñoz and Losos (2018), temperature was recorded every 10 min during a 4-h trial, and T_pref_ was estimated as the mean of the central 50% of temperatures measured during the experiment.

We estimated the relationships between each physiological trait (CT_min_, T_pref_, and CT_max_) and elevation using phylogenetic generalized least squares analysis using the *gls* function in the R package caper (Orme et al. 2013) and the *Anolis* phylogeny provided by Poe et al. (2017), which we pruned down to our taxa of interest. We simultaneously estimated phylogenetic signal (λ) in the residual error with the regression parameters (Revell 2010). We performed multiple ANCOVAs (analysis of covariance; treating each physiological trait as a fixed effect) to determine whether the relationship between within-population trait range and elevation differed among traits, such that it was higher in T_pref_ and CT_max_. All statistical analyses were run in R (R Development Core Team 2014).

**Fig. 3:**
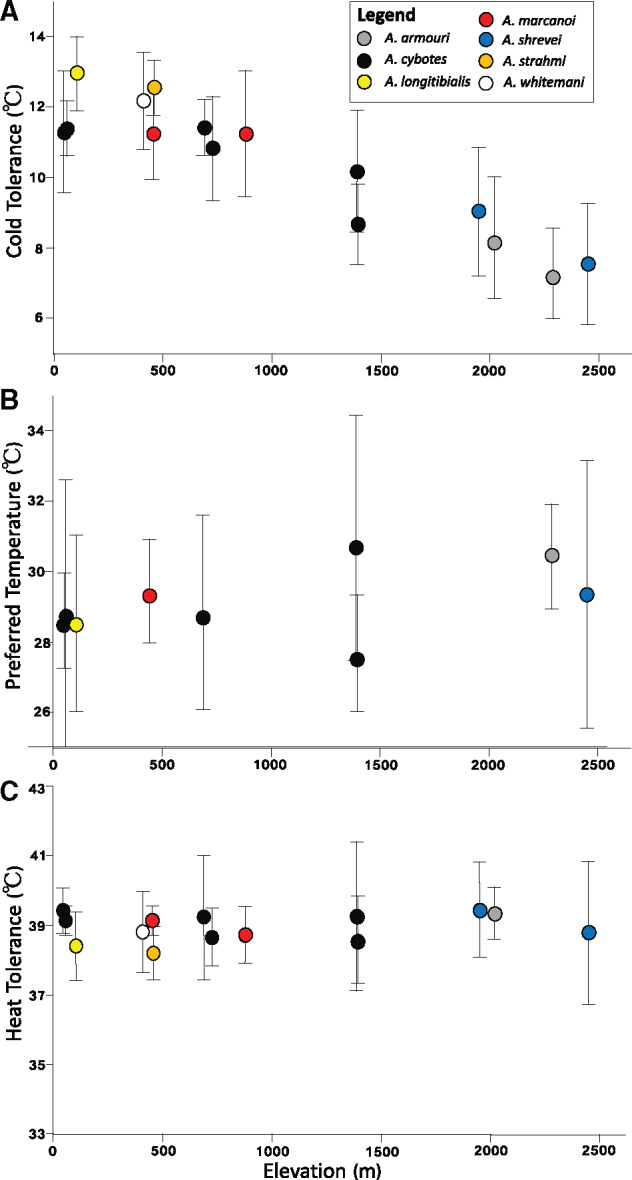
Population means (±1 SD) for (**A**) cold tolerance, (**B**) the preferred temperature, and (**C**) heat tolerance are given, with elevation provided on the *x*-axis. Circle color and shape denote species identity, following the legend provided.

**Fig. 4:**
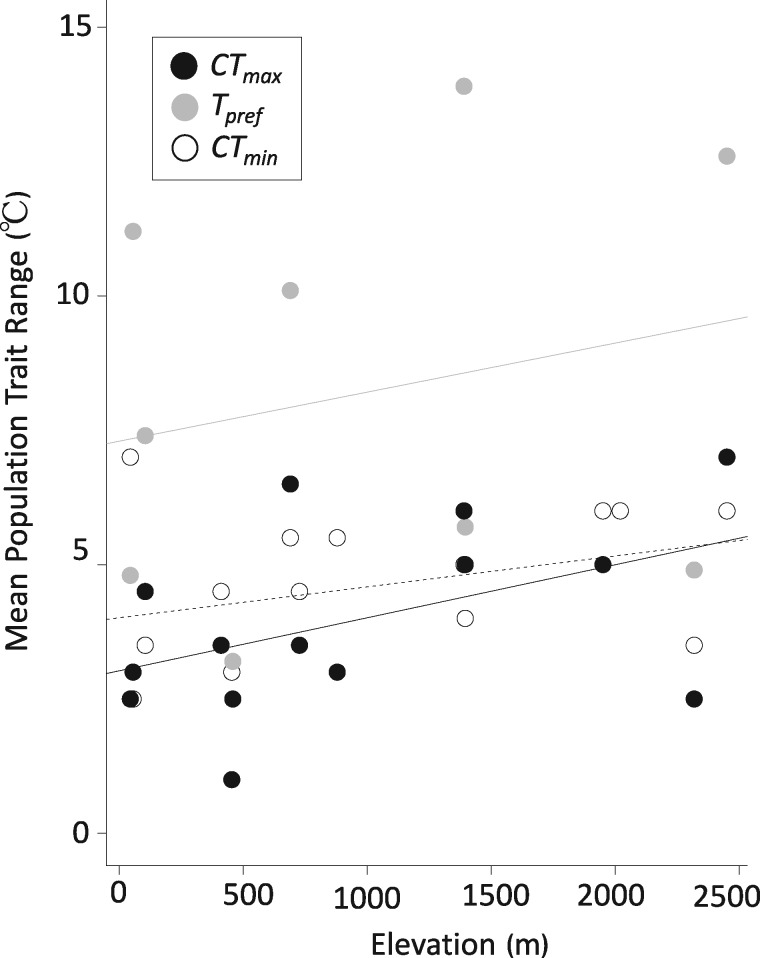
Population ranges for physiological traits are given, with circle color denoting species identity following the legend provided. The gray line shows the relationship between altitude and trait range for T_pref_. The black line shows the relationship between altitude and trait range for CT_max_. The dashed line shows the relationship between altitude and trait range for CT_min_.

We found that mean cold tolerance increased with elevation, such that lizard populations near the mountaintop were more resistant to low temperatures than their counterparts near sea level (Adj. *R*^2^ = 0.990, *P *<* *0.001) (Fig. 3A). Specifically, mean CT_min_ dropped from 12°C to 13°C in populations at low elevations to ∼7.5°C in populations from high elevations, corresponding to a decline of ∼0.3°C per 100-m increase in altitude. In contrast to CT_min_, neither mean CT_max_ (Adj. *R*^2^ = 0.092, *P *=* *0.092) nor mean T_pref_ (Adj. *R*^2^ = 0.153, *P *=* *0.153) shifted with elevation (Fig. 3B, C). Mean CT_max_ ranged between 38°C and 39°C for all populations, regardless of elevation. Mean T_pref_ was similarly narrow, and ranged between 28°C and 31°C for all populations, regardless of elevation. Although T_pref_ showed no relationship with elevation, variance for this trait was significantly higher than for CT_min_ (*F*_1,22_=9.94, *P *=* *0.005) and CT_max_ (*F*_1,21_=14.58, *P *=* *0.001). At any given elevation, within-population trait range was considerably higher for T_pref_ than for CT_min_ or CT_max_ (fixed effect coeff: 4.2 ± 1.0, *P *<* *0.001; Fig. 4).

Taken together, our results suggest an imperfect relationship between thermal environment and physiological adaptation. On the one hand, within-population variation was low for CT_min_, and this trait shifted strongly with elevation, which could reflect narrow nighttime variation and low thermal overlap across elevation. Within-population variation in T_pref_ was significantly higher than in CT_min_, consistent with broader variation in daytime temperatures, and consistent with previous work finding low repeatability in this trait (Clusella-Trullas et al. 2007). On the other hand, neither T_pref_ nor CT_max_ shifted with elevation, and within-population variation for CT_max_ is as low as for CT_min_.

Of key consideration is that minimum and maximum environmental temperatures, while likely important, are certainly not the only factors shaping physiological evolution. For example, several studies report low heritability for physiological traits (Logan et al. 2018; Martins et al. 2018), and other studies have found that heat tolerance is particularly unresponsive to experimental selection (e.g., Gilchrist and Huey 1999; Baer and Travis 2000; Barrett et al. 2011; Doyle et al. 2011, but see Huey et al. 1991; Santos et al. 2012). These factors may contribute to high levels of conservatism often observed in heat tolerance (Kellermann et al. 2012b; Araújo et al. 2013). Furthermore, plasticity may relate more to local environment than species’ means in physiological traits (e.g., Hoffmann et al. 2013). Physiological traits are often subject to different underlying biochemical constraints. Because biochemical reactions accelerate with temperature (up until an upper limit), maximum performance, metabolism, and growth should increase with higher body temperatures (Hamilton 1973; Bennett 1987). This “hotter is better” hypothesis supplies a potential mechanism for why upper physiological traits should remain high even in cold environments, and why variance surrounding CT_max_ might be low (Angilletta et al. 2010). It is also important to note that the selective milieu influencing heat and cold tolerance likely differ substantially from each other, and that this may factor into how their evolution may be compared (DeWitt 1967; DeWitt and Friedman 1979). As will be discussed in the next section, another possibility, which is not mutually exclusive with the mechanisms described above, is that thermoregulatory behavior has different effects on upper and lower physiological traits.

## Thermoregulatory behavior and its impacts on thermal physiology

Physiological processes are strongly dependent on temperature in ectotherms, and are typically optimized within a relatively narrow range of body temperatures (Huey 1982; Angilletta et al. 2002; Angilletta 2009). Organisms can use behavior to preferentially select portions of the habitat that more closely match their optimal range (Stevenson 1985; Hertz et al. 1993). By adjusting their shade use, activity times, or even simply their posture, ectotherms such as lizards can much more narrowly restrict the range of temperatures in their habitats that they actually experience (Cowles and Bogert 1944; Bartholomew 1966; Huey and Pianka 1977; Stevenson 1985; Kearney et al. 2009). Thus, through behavior, organisms can mold both the mean and variance of the thermal conditions they experience. The consequence of behavioral regulation is that lizards can substantially homogenize temporal and spatial variation in their thermal environments.

Thermoregulation requires thermal heterogeneity: in order for behavioral adjustments to be effective, sufficient variation must be present in the habitat and transit distances between thermal patches should be relatively low (Huey 1974; Hertz 1992b; Sears and Angilletta 2015). Otherwise, the costs associated with regulating temperature might exceed the potential benefits of a higher, stable core temperature. Given that thermal variation is substantially higher during the day, behavioral thermoregulation could contribute to the low variation in preferred temperature and critical thermal maximum across elevation. Janzen (1967) recognized the potential for behavior to alter the range of conditions organisms experience, stating “by regulating its activity, (an organism) places itself in a more uniform environment during major activity periods.” As a consequence, regulatory behavior has the potential to “flatten” the physiological barriers imposed by altitudinal shifts in ambient temperature (Buckley et al. 2013).

**Fig. 5:**
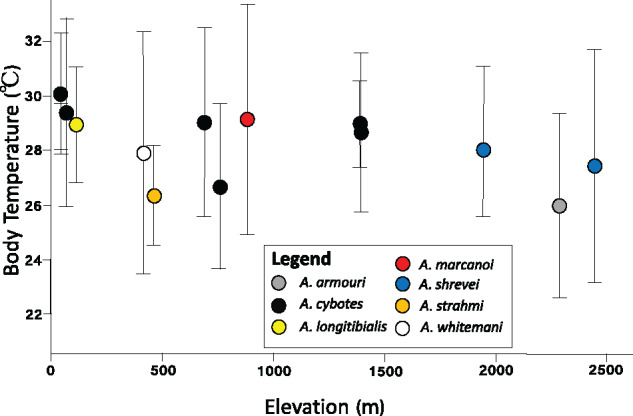
Population means (±1 SD) for body temperature are given, with elevation provided on the *x*-axis. Circle color and shape denote species identity, following the legend provided.

At the same localities where physiological traits were measured, we also recorded body temperature for field-active lizards during a single day (0600–1900) of sampling (Muñoz et al. 2014). For each lizard captured, we recorded body temperature using a type T thermocouple, and recorded the lizard’s basking behavior (i.e., whether it was using a shaded perch, a sunlit perch, or a semi-shaded perch) and microhabitat use (i.e., tree trunk, boulder, bare ground, etc.). Body temperature data indicated that, indeed, lizards behaviorally thermoregulate (Fig. 5). Despite living in environments that, on average, differ by 15°C, mean body temperature ranged between 26°C and 30°C (Supplementary Table S1). We further found that effective thermoregulation is driven largely by altitudinal shifts in shade use: whereas lowland lizards were nearly always found in the shade, basking behavior increased with elevation, such that lizards found above 2000 m were nearly always observed basking in the sun (Muñoz et al. 2014). Concurrently, montane lizards also shifted their structural microhabitat use to boulders (as opposed to the preferred arboreal habitat of lowland cybotoids), as boulders were substantially warmer perches than tree trunks and branches (Hertz and Huey 1981; Muñoz and Losos 2018). This habitat switch allowed high elevation lizards to maintain core temperatures very close to that of their low elevation counterparts, and facilitated highly precise thermoregulation (Muñoz and Losos 2018). Given that maximal performance often positively correlates with core temperature (Kingsolver and Huey 2008; Angilletta et al. 2010), montane lizards may thermoregulate to capitalize on the thermodynamic advantages of higher body temperatures. Thermal homogeneity at night, in contrast, likely precludes fine-scale thermoregulation (but see Webb and Shine 1998; Anderson et al. 2005; Rock and Cree 2008), which may have contributed to the pronounced structuring of cold tolerance across elevation.

## Thermal variability, behavioral thermoregulation, and the Bogert effect

By restricting the thermal variation that they experience, thermoregulating organisms are the architects of their own selective environments (Odling-Smee et al. 1996; Laland et al. 2017). Regulatory behaviors shield organisms from environmental extremes, thus buffering them from directional selection on physiology and, potentially, precluding the need to evolve even in the face of changing environmental conditions. As a consequence, physiological evolution should be slowed when behavioral buffering occurs (Huey et al. 2003). This idea—that thermoregulation constrains physiological evolution—was initially proposed by Bogert (1949), after many years spent observing behavioral thermoregulation in North American reptiles. As a homage to Bogert’s pioneering efforts, Huey et al. (2003) coined this phenomenon (behavioral buffering of selection on physiology) as the Bogert effect.

In their original paper describing the Bogert effect, Huey et al. (2003) used a null-model approach to illustrate how thermal physiology would respond to altitudinal shifts in thermoregulating and non-thermoregulating lizards. Specifically, when organisms thermoregulate, physiology should shift little (or not at all) with elevation, whereas clinal physiological divergence is expected when organisms do not thermoregulate. Consequently, physiological evolution should be slower in thermoregulating lizards. The same logic can be applied to different traits within an organism by considering the ways in which thermal heterogeneity differently impacts thermoregulatory efficiency for different physiological traits. As illustrated above, daytime thermal heterogeneity should facilitate thermoregulation, whereas nighttime temperatures are stable and become progressively cooler with elevation (Fig. 2). By extension, buffering behaviors should limit the evolution of heat tolerance and the preferred temperature, while facilitating evolution in cold tolerance.

To test this hypothesis, we compared the Brownian motion estimate of the evolutionary rate parameter (σ^2^) among the three physiological traits. Specifically, we used likelihood ratio tests (LRTs) to compare evolutionary rates among traits (Adams 2013). We compared the likelihood a model in which rates of evolution were constrained to be the same among traits (e.g., σ^2^_CTmin_ = σ^2^_T__pref_) to a model in which rates were allowed to vary (e.g., σ^2^_CTmin_ ≠ σ^2^_T__pref_). We bounded our rate estimates using 95% confidence interval, which we derived from the standard error as estimated from the square root diagonals of the inverse Hessian matrix. D. Adams (pers. comm.) supplied custom code for this function.

**Fig. 6:**
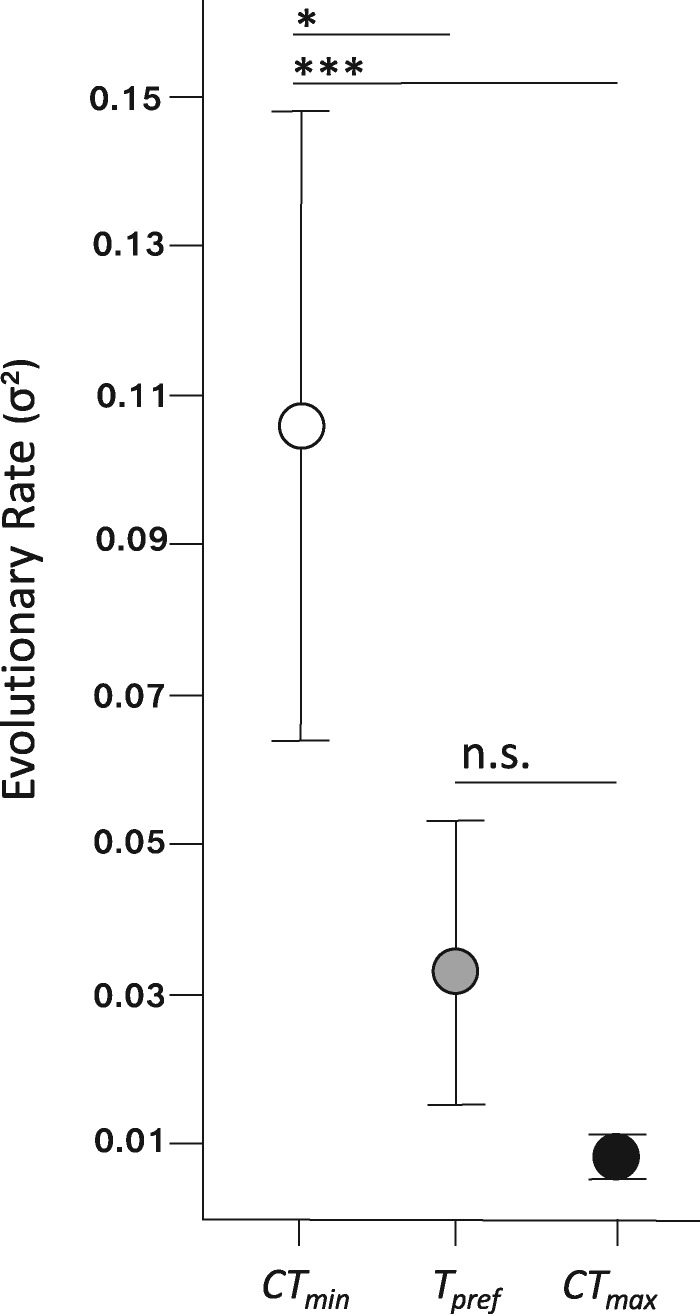
The rate of evolution is an order of magnitude faster for cold tolerance (CT_min_) than for the preferred temperature (T_pref_) and heat tolerance (CT_max_). Bars represent the 95% confidence interval. Bars show significance values for differences in evolutionary rate, as follows: * *P *<* *0.05; *** *P *<* *0.001; n.s.: not significant.

CT_min_ does, indeed, evolve faster than both T_pref_ (LRT = 6.4, p = 0.012) and CT_max_ (LRT = 12.4, *P* < 0.001) (Fig. 6; Muñoz et al. 2014). In contrast, rates of evolution were similar between T_pref_ and CT_max_ (LRT = 0.3, *P* = 0.578). Our results point to general principles that may link environmental heterogeneity and behavior with the tempo of physiological evolution. Specifically, when resources are broad and shared across habitats, thermoregulation has the potential to buffer organisms from selection, resulting in physiological stasis despite environmental variation. In contrast, environmental structuring should limit behavioral buffering and therefore result in faster physiological evolution. Thus, we suggest that thermoregulatory behavior, mediated by thermal resource availability, is one of the key (though not exclusive) factors influencing patterns of physiological divergence and rates of evolution. Theoretically, these phenomena could encompass any number of abiotic or biotic variables, not just temperature. For example, environmental and behavioral variation may impact different patterns of hydric physiology within and among species of Appalachian salamanders, and in turn impact physiological rates of evolution (Gifford 2016).

As a caveat, however, environmental heterogeneity is not the only factor that determines whether or not (or how well) organisms can thermoregulate. All regulatory behaviors are subject to costs. For example, thermal habitat quality (the available of suitable temperatures) may be too low for effective thermoregulation (Huey 1974; Huey and Slatkin 1976; Hertz et al. 1993), or the spatial structuring of suitable temperatures may preclude effective thermoregulation (Hertz 1992b, Sears et al. 2015). Moreover, investment in thermoregulation can impose costs to fitness, for example, by diverting time from foraging and diverting energy from growth (Sears 2005; Brewster et al. 2013). Basking behavior can also expose organisms to predators: for example, high elevation cybotoids are warier than their low elevation counterparts, perhaps in response to spending much of their time basking in exposed areas (Boronow et al. 2018). As such, the environmental conditions for thermoregulation could be present without behavioral buffering being favored.

Findings from available meta-analyses generally find that upper physiological limits are more conserved within lineages and across environments than cold tolerance (Araújo et al. 2013; Hoffmann et al. 2013; Sunday et al. 2014). Finer-scale studies within select clades also find that heat tolerance is generally more evolutionarily inert than cold tolerance (Kellermann et al. 2012b; Muñoz et al. 2016; but see Diamond et al. 2017). Combined with the performance benefits of higher core temperatures (reviewed in Angilletta et al. 2010), these results suggest that thermoregulation may help limit evolution of upper physiological limits, while lower physiological limits correlate with local environmental conditions. Thus, the connection between diurnal temperature variation and behavioral thermoregulation may impart predictable macroevolutionary signatures on physiology across ectotherms.

Behavioral thermoregulation can help explain why heat tolerance and the preferred temperature evolve more slowly than cold tolerance, but not why these traits are not even higher in lowland populations. Although CT_max_ hovered around 39–40°C in the cybotoids, heat tolerance approaches 50°C in several lizard species (e.g., Araújo et al. 2013) and operative temperatures measured at low elevation often exceeded 40°C. If the “hotter-is-better” hypothesis is true, then it is not clear why upper physiological limits, particularly in lowland habitats, are not higher. It is possible that extremely hot temperatures are less frequently available in lizards’ environments (a point we cannot address here), that phylogenetic constraints limit heat tolerance evolution in tropical anoles, or that any number of other selective constraints (described above) stymie the evolution of upper thermal limits.

## Concluding remarks

The goal of this study was to connect climate variation, behavioral thermoregulation, and physiological evolution in a common conceptual framework. Janzen’s predictions for climatic variability served as the springboard for discussion, with focus here on daytime and nighttime temperature variation. Theoretically, however, Janzen’s predictions should extend beyond temperature to other physical characteristics of the environment (e.g., acidity, precipitation, salinity, light environment, and oxygen concentration). Janzen (quoted in Sheldon et al. 2018) recognized this, stating “mountain passes are higher in the tropics from the viewpoint of the physiological animal, and therefore montane barriers are greater in the tropics. Needless to say, the concept applies to any organism *vis-à-vis the milieu in which it is situated*” (emphasis added). In other words, the amount of fluctuation in any resource should be positively correlated with an organism’s tolerance to that resource. The work described here is not the first to make such extrapolations. For example, a similar parallel has been drawn between arboreal and terrestrial thermal habitats, which, when compared across elevation, mimic thermal regimes observed by Janzen (1967) across seasons and latitude (Scheffers et al. 2017).

Janzen’s (1967) hypothesis and the Bogert effect independently provide important perspectives on the mechanisms shaping physiological diversity (Huey et al. 2003; Ghalambor et al. 2006; Sheldon et al. 2018). By connecting these concepts, we can elucidate the interactions between climatic variation and behavior in shaping rates of physiological evolution. As these patterns extend beyond temperature to incorporate other physical and biotic resources, the connections presented here may be widespread and may shed important insight on the factors that guide the rate and pattern of evolutionary change.

## Supplementary data

Supplementary data is available at *Integrative Organismal Biology* online.

## Supplementary Material

Supplementary Data
