# Supplementary material for: Janzen’s Hypothesis Meets the Bogert Effect: Connecting Climate Variation, Thermoregulatory Behavior, and Rates of Physiological Evolution
Source: Integr Org Biol. 2019 Jan 2;1(1):oby002. doi: 10.1093/iob/oby002 (PMC7671085; doi:10.1093/iob/oby002)
Supplement: Supplementary Data [file oby002_supp.docx]

**Supplementary Information** Map **Figure S1.** Map of Hispaniola showing topographic variation across the island. The Cordillera Central is the large mountain range in the central-northern portion of the island. The Sierra de Baoruco is the mountain range in the south-southwestern portion of the island. Each point shows a sampling locality for a cybtoid species. The phylogenetic relationships for the cybotoids are given, as the inset shows the location of Hispaniola within the Caribbean. Map provided by V. Farallo.

**Figure S2.** Copper lizard models deployed on (A) boulders and (B) tree trunks. Photos taken from Valle Nuevo (elevation = 2,450 m) in the Cordillera Central of the Dominican Republic. Photos provided by the author.

**Table S1**. Data from Muñoz et al. (2014) showing locality information (latitude, longitude, altitude, mean annual temperature) for each population of lizard sampled. Mean body temperature ± 1 sd is given, with sample size provided in parentheses. Populations of *Anolis cybotes* with SB in parentheses denote populations from the Sierra de Baoruco mountain chain whereas those with a CC in parentheses come from the Cordillera Central mountain chain. Asterisks denote localities where operative temperature data were taken (data presented in Figure 2). *T_pre_*_f_ data for *A. marcanoi* were gathered from a nearby locality (18.24, -70.26, elevation = 422 m).

| **Species** | **Latitude** | **Longitude** | **MAT (ºC)** | **Alt. (m)** | ***T_b_* (ºC)** |
| --- | --- | --- | --- | --- | --- |
| *A. cybotes* (SB)* | -71.19 | 17.96 | 24.8 | 45 | 30.1 ± 2.3 (45) |
| *A. cybotes* (CC) | -70.13 | 18.42 | 26.0 | 56 | 29.4 ± 3.5 (53) |
| *A. longitibialis* | -71.45 | 17.8 | 25.8 | 105 | 28.9 ± 2.2 (101) |
| *A. whitemani* | -71.57 | 18.32 | 24.3 | 411 | 27.9 ± 4.5 (17) |
| *A. strahmi* | -71.62 | 18.34 | 22.3 | 454 | 26.3 ± 1.9 (7) |
| *A. marcanoi* | -70.5 | 18.55 | 23.4 | 458 | - |
| *A. cybotes* (CC) | -70.61 | 19.1 | 22.4 | 690 | 29.0 ± 3.5 (39) |
| *A. cybotes* (SB)* | -71.14 | 18.06 | 23.1 | 727 | 26.6 ± 3.1 (53) |
| *A. marcanoi* | -70.54 | 18.65 | 19.6 | 879 | 29.1 ± 4.3 (48) |
| *A. cybotes* (CC) | -70.73 | 18.86 | 17.2 | 1390 | 29.2 ± 1.6 (10) |
| *A. cybotes* (SB)* | -71.28 | 18.18 | 18.0 | 1395 | 28.6 ± 3.0 (11) |
| *A. shrevei* | -70.7 | 18.84 | 13.7 | 1950 | 28.0 ± 3.1 (10) |
| *A. armouri* | -71.7 | 18.3 | 13.3 | 2020 | - |
| *A. armouri** | -71.71 | 18.29 | 11.4 | 2318 | 25.9 ± 3.4 (21) |
| *A. shrevei* | -70.6 | 18.73 | 10.1 | 2450 | 27.4 ± 4.4 (20) |
